# Differential expression and clinical significance of long non-coding RNAs in the development and progression of lung adenocarcinoma

**DOI:** 10.3389/fonc.2024.1411672

**Published:** 2024-06-06

**Authors:** Haitao Wei, Sa Zhang, Xiaojin Lin, Ruirui Fang, Li Li

**Affiliations:** ^1^ Huaihe Hospital of Henan University, Kaifeng, Henan, China; ^2^ Institute of Nursing and Health, Henan University, Kaifeng, Henan, China

**Keywords:** long non-coding RNAs, lung adenocarcinoma, differential expression, diagnostic markers, prognostic marker, clinical application, signal pathways

## Abstract

With the development of gene testing technology, we have found many different genes, and lncRNA is one of them. LncRNAs refer to a non-protein coding RNA molecule with a length of more than 200bp, which is one of the focuses of research on human malignant diseases such as LUAD. LncRNAs act as an oncogene or inhibitor to regulate the occurrence and progression of tumors. The differential expression of LncRNAs promotes or inhibits the progression of lung adenocarcinoma by affecting cell proliferation, metastasis, invasion, and apoptosis, thus affecting the prognosis and survival rate of patients. Therefore, LncRNAs can be used as a potential target for diagnosis and treatment of cancer. The early diagnosis of the disease was made through the detection of tumor markers. Because lung adenocarcinoma is not easy to diagnose in the early stage and tumor markers are easy to ignore, LncRNAs play an important role in the diagnosis and treatment of lung adenocarcinoma. The main purpose of this article is to summarize the known effects of LncRNAs on lung adenocarcinoma, the effect of differential expression of LncRNAs on the progression of lung adenocarcinoma, and related signal transduction pathways. And to provide a new idea for the future research of lung adenocarcinoma-related LncRNAs.

## 1 Introduction

In the past, much of the non-protein-coding portion of the human genome has historically been regarded as useless DNA. Through examination, we found different classes of non-coding RNAs. Non-coding RNAs are broadly categorized based on their size: small non-coding RNAs represent noncoding transcripts less than 200 nucleotides in length, such as miRNAs, siRNAs, and piRNAs (1). Among the various types of non-protein-coding transcripts, a class referred to as long noncoding RNAs (lncRNAs) has attracted increasing attention. LncRNAs are defined as transcripts of more than 200 nucleotides that are not translated into proteins (2). LncRNAs are often master regulators of gene expression and exert their functions through transcriptional, post-transcriptional, and epigenetic gene-regulatory mechanisms (3). For the past few years, LncRNAs have become one of the hotspots of tumor research, and the dysfunction of LncRNAs is considered to be one of the main factors leading to a variety of diseases, including cancer, cardiovascular disease, fibrosis, obesity, etc. (4). LncRNAs are involved in oncogenes and tumor suppressor genes by regulating target genes or signal pathways at epigenetic, transcriptional and post-transcriptional levels (5).

Increasing studies demonstrated the involvement of thousands of LncRNAs in almost all life processes and played an indispensable regulatory role. Studies have found that there are two broad mechanisms by which LncRNAs exert their function. One mode of function involves LncRNA associations with promoters and regulatory regions of genes, where they recruit transcriptionally-activating or repressive protein complexes that are either directly involved in transcriptional initiation, or alternatively chromatin-remodeling complexes that once recruited by LncRNAs render chromatin transcriptionally repressive. Another mode of function involves LncRNAs that act by sequestration of regulatory factors. One example is that of “sponge” LncRNAs (ceRNAs) that bind microRNAs, thus protecting their target mRNAs from the effects of microRNAs. Since microRNAs typically repress translation or destabilize mRNAs, LncRNA sponge action typically increases protein production. A second example of sequestration-acting LncRNAs is that of decoy LncRNAs, which sequester gene regulatory proteins (2). Some LncRNAs were spliced into mRNA-like PolyA tail and promoter structures, which could be dynamically expressed and spliced in different ways during tissue and organ differentiation, thus showing time and space specificity.

Meantime, about LncRNA functions, many studies have found that LncRNAs have multiple biological functions: they are involved in chromatin modification, genomic imprinting, intranuclear transportation, and other regulations. Protecting protein-coding genes in multiple modes; containing highly efficient key sequences, and more sensitive structural and functional constraints than proteins; having conserved secondary structure, splicing form, and precise subcellular localization; having cis and trans regulation. The function of LncRNAs cannot be determined by its simple classification but is usually determined by its localization, sequence, and/or secondary structure.

In LncRNA expression, further development of genomic technologies has proved that LncRNAs are important in complex organisms by regulating chromatin remodeling and transcriptional regulation, etc., and participate in the pluripotency and differentiation of stem cells, which correlate to the progression of a variety of human diseases like cancer and neurological disorders. Different LncRNAs play different roles in cellular processes. Some LncRNAs participate in the imprinting process, affecting the single allelic expression of genes. Some LncRNAs perform epigenetic modification by introducing chromatin remodeling complexes into specific chromatin sites (6).

## 2 LUAD overview

Lung cancer is the second most common malignancy in the world and the death rate is increasing year by year (7, 8). Lung cancer is mainly divided into small cell (SCLC) and non-small cell (NSCLC). Approximately 80–85% of lung patients are diagnosed with non-small cell lung cancer (NSCLC) in pathological diagnosis, of which lung adenocarcinoma (LUAD) is the most common subtype (9, 10). Lung adenocarcinoma (LUAD) is the most common histologic type of lung cancer with a high mortality rate. LUAD is one of the deadliest cancers in the world. The main treatment for LUAD is surgery, but the prognosis is poor due to Histological heterogeneity (11). LUADs originate from cells that secrete surfactant components. Morphological patterns of LUADs include acinar, papillary, solid, micropapillary, and invasive mucinous types. Lepidic components or pure lepidic patterns are found in noninvasive forms, previously classified as bronchoalveolar carcinoma, which can be associated with invasive mucinous or acinar LUAD (12). As for clinicopathological features of lung cancer, the most common is smoking. However, LUAD can occur in both smokers and non-smokers. LUADs are the most common type of lung cancer seen in non-smokers. It is more common in women than in men and is more likely to occur in younger people than other types of lung cancer and to present at more advanced stages of the disease (13).

Early symptoms of lung cancer are relatively benign, and this contributes to low detection rates in early disease stages. And because the lung adenocarcinoma is not sensitive to the treatment of anticancer drugs, the drug resistance increases gradually, and the drug resistance is not obvious. Therefore, the metastatic rate of advanced lung cancer is high and the overall prognosis is poor (14). For the past few years, despite the advancement of the treatment for LUAD, it still has some limitations on the whole. The early diagnosis of LUAD is difficult, and it tends to relapse and metastasis, leading to poor prognosis for LUAD patients. Although great progress has been made in diagnosis and treatment recently, the long-term clinical prognosis and overall survival of lung adenocarcinoma (LUAD) patients remain unsatisfactory (15). The inadequate comprehension of LUAD-related biological mechanisms limits the improvement in therapeutic efficacies. Thus, it is urgent to further elucidate the involved mechanisms of cancer progression to improve the clinical outcome of LUAD patients (16).

## 3 The mechanism of LncRNAs in LUAD

With the development of next-generation sequencing technology, the in-depth study of non-coding genes has attracted more and more attention from researchers. Many studies have shown that the treatment of tumors is related to LncRNAs. Aberrantly expressed LncRNAs play pivotal roles in the progression of LUAD. In the meantime, the aberrant expression and mutation patterns are tightly related to tumor proliferation, apoptosis, invasion, metastasis, TNM stage, drug resistance, lymphatic metastasis, and shorter prognosis. For example, Zhang et al (17) found that the up-regulate of CRNDE was significantly correlated with poor differentiation, TNM stage, lymph node metastasis, radiotherapy response, and significantly shorter overall survival. In addition to the abnormal expression of LncRNAs has an impact on lung adenocarcinoma, some LncRNAs can also act as oncogenes to interact with miRNA, thus acting with certain proteins to affect the function of lung adenocarcinoma cells. MicroRNAs (miRNAs) are conserved, non-coding short RNA molecules. MiRNAs can regulate numerous cellular functions such as cell growth, migration, invasion, and EMT (18). Significant evidence shows that abnormal regulation of miRNAs is related to the malignant progression of many cancers, and miRNAs are potential therapeutic targets for lung cancer (19). Yang et al (14) found that LncRNA PCAT6 can act as a molecular sponge for miR-545-3p and that this promotes LUAD development.

## 4 The differential expression of lncRNA in LUAD

Long non-coding RNAs (LncRNAs) are a kind of transcriptional RNA containing over 200 nucleotides, which are encoded by the genome and lack protein-coding function. It is related to a variety of cellular processes, including the regulation of gene expression (including epigenetics, pre-transcription, and post-transcription), tissue metabolism, cell growth, differentiation, and development (20). The abnormal expression of LncRNAs is significant in multiple cancers.

**Figure 1:**
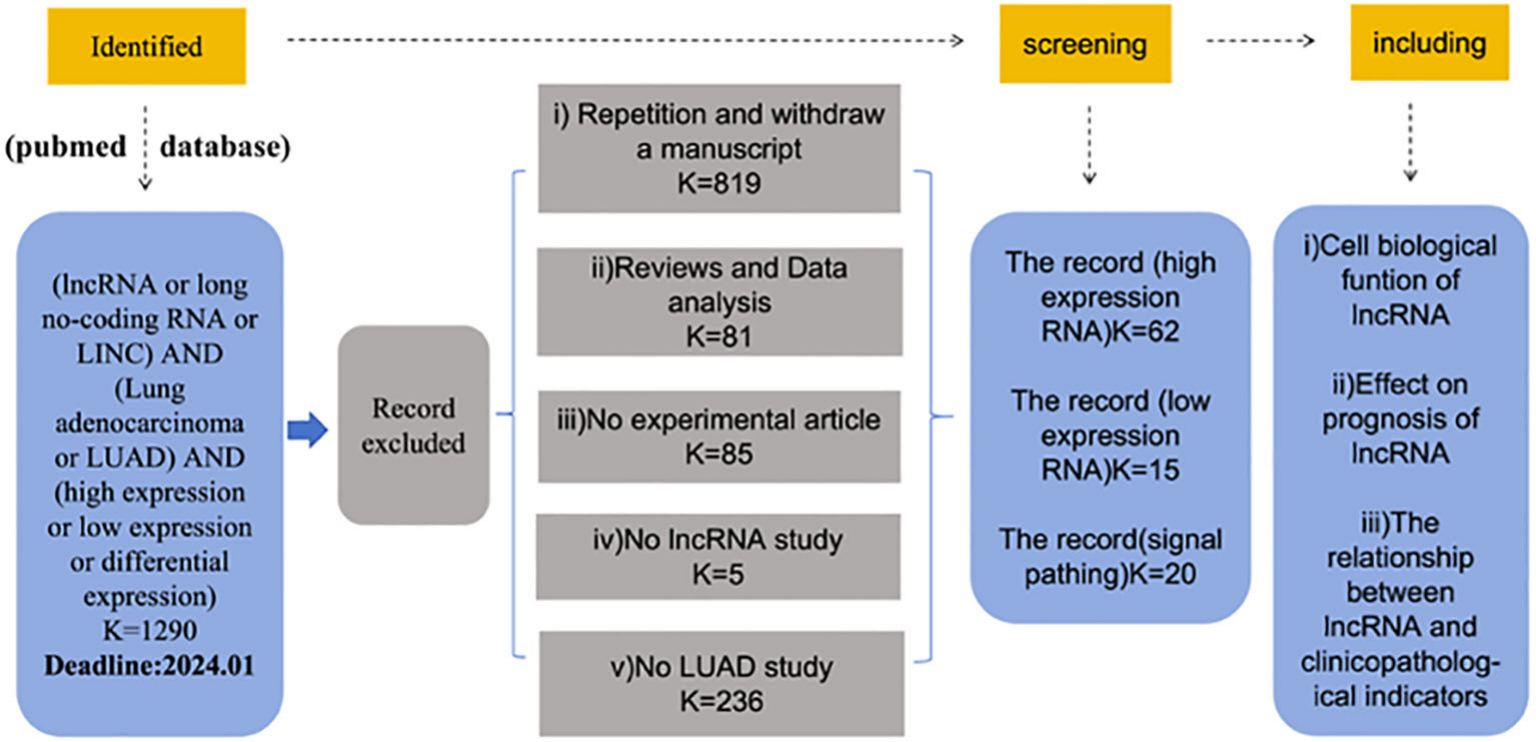
Screening of differentially expressed lncRNA in lung adenocarcinoma.

To distinguish the published LncRNAs biomarkers for diagnosis or prognosis in lung adenocarcinoma patients, entry terms “(LncRNA OR long no-coding RNA) AND (lung adenocarcinoma OR LUAD) AND (high expression OR low expression)” were firstly seek in the NCBI PubMed database. Finally,62 up-regulated LncRNAs and 15 down-regulated LncRNAs in lung adenocarcinoma were identified from these studies, as shown in **Figure 1**.

### 4.1 High-level expressed lncRNA in LUAD and clinical significance

During the data retrieval, we found that 62 articles demonstrated the up-regulation of LncRNAs. When analyzing these data, we found that the up-regulation of LncRNAs is often related to the poor prognosis of lung adenocarcinoma, but also affects the function of lung adenocarcinoma cells. At the same time, we also found that the clinicopathology of most LncRNAs is related to tumor size, TNM stage, and lymph node metastasis. The data are shown in **Table 1**.

**Table 1:** Biological function and clinical significance of up-regulated lncRNA in lung adenocarcinoma.

Order (high expression)	Pathology type	Sample	Expression	Clinicopathological parameters associated	Cell biological function	Prognosis	PMID	DOI
LncRNA A2M-AS1	LUAD	Tissues Cell	U		cell proliferation, migration , invasion、 growth	Poor prognosis	**36461613**	10.1177/09603271221138971
LncRNA BC	LUAD	Tissues Cell Animals	U		Cell migration、proliferation, epithelial-mesenchymal transition (EMT)	Poor prognosis	**36650118**	10.1002/ctm2.1129
LncRNA CASC11	LUAD	Tissues	U	TNM stage, tumor size, and lymph node metastasis	cell proliferation,	Poor prognosis	**36705085**	10.2217/pme-2022-0104
LncRNA CYTOR	LUAD	Cell Tissues Animals	U		gemcitabine resistance and EMT	Poor prognosis	**38273783**	10.3724/abbs.2023287
LncRNA CASC9.5	LUAD	Tissues Cell Animals	U	TNM stage, tumor size, and lymph node metastasis	cell proliferation, migration	Poor prognosis	**29311567**	10.1038/s41598-017-18280-3
LncRNA CAR10	LUAD	Tissues Cell Animals	U		cell proliferation, invasion, migration、epithelial-mesenchymal transition(EMT)	Poor prognosis	30617305	10.1038/s41388-018-0645-x
LncRNA CRNDE	LUAD	Tissues Cell	U	poor differentiation, TNM stage, lymph node metastasis, radiotherapy response, and a significantly shorter overall survival.	cell proliferation, apoptosis.	Poor prognosis	**28550688**	10.3727/096504017X14944585873668
LncRNA DANCR	LUAD	Tissues Cell	U		cell migration invasion	Poor prognosis	**30535487**	10.3892/or.2018.6897
LncRNA DANCR	LUAD	Tissues Cell Animals	U		cell proliferation, migration and invasion and cell apoptosis	Poor prognosis	**29266795**	10.1111/jcmm.13420
LncRNA FAM207BP	LUAD	Tissues Cell	U		cell proliferation, migration, invasion	Poor prognosis	**33434533**	10.1016/j.lfs.2021.119022
LncRNA FAM83A-AS1	LUAD	Tissues Cell Animals	U	tumor size, lymph node metastasis, and TNM stage.	cell proliferation, migration, invasion	Poor prognosis	31522616	10.1080/15384101.2019.1664225
LncRNA FAM83A-AS1	LUAD	Tissues Cell Animals	U		cell migration, invasion and growth	Poor prognosis	**36960858**	10.3892/or.2023.8532
LncRNA FAM83A-AS1	LUAD	Tissues Cell Animals	U		cell proliferation and metastasis	Poor prognosis	**33687144**	10.1111/1759-7714.13928
LncRNA FBXL19-AS1	LUAD	Cell Animals	U	tumor size、TNM stage, and lymph node metastasis.	cell proliferation, migration	Poor prognosis	31566718	10.1002/jcp.29251
LncRNA FAM230B	LUAD	Tissues	U	tumor size	cellular viability, migration and invasion for LUAD cells	Poor prognosis	**35287554**	10.1080/21655979.2022.2034568
LncRNA GLIDR	LUAD	Cell	U		cell proliferation, apoptosis	Poor prognosis	**34369267**	10.1080/15384101.2021.1953754
LncRNA HCP5	LUAD	Tissues Cell Animals	U		cell proliferation, invasion,	Poor prognosis	31131047	10.7150/thno.31097
LncRNA H19	LUAD	Tissues Cell Animals	U	Tumor size and tumor weight	cell proliferation, invasion, migration,	Poor prognosis	31317666	10.1111/jcmm.14533
LncRNA HCG18	LUAD	Tissues Cell Animals	U		cell proliferation, migration , invasion、growth	Poor prognosis	**32559619**	10.1016/j.biopha.2020.110217
LncRNA HOTTIP	LUAD	Tissues Cell	U	Drug resistance	cell proliferation,	Poor prognosis	**29272003**	10.26355/eurrev_201712_14013
LncRNA HNF1A-AS1	LUAD	Tissues Cell Animals	U	TNM stage, tumor size, and lymph node metastasis	cell proliferation, migration and epithelial-mesenchymal transition (EMT)	Poor prognosis	**25863539**	10.18632/oncotarget.3247
LncRNA KCNQ1OT1	LUAD	Tissues Cell	U	tumor size, poor differentiation, positive lymphatic metastasis and high TNM stages.	cell Proliferation, chemotherapy resistance	Poor prognosis	28600629	10.1007/s00280-017-3356-z
LncRNA LOXL1-AS1	LUAD	Tissues Cell	U		cell proliferation, migration, apoptosis	Poor prognosis	31758653	10.1002/cam4.2641
LncRNA LOC389641	LUAD	Tissues Cell	U	poor patient survival	cell proliferation, apoptosis , invasion, colony formation	Poor prognosis	**33318313**	10.18632/aging.202286
LncRNA LOC107985872	LUAD	Cell Animals	U		cell migration invasion and epithelial-mesenchymal transition (EMT)	Poor prognosis	**32829169**	10.1016/j.envpol.2020.115307
LncRNA LEISA	LUAD	Tissues Cell Animals	U	shorter overall survival times in LAD patients	cell proliferation, apoptosis	Poor prognosis	**33859372**	10.1038/s41388-021-01769-7
LncRNA MIR31HG	LUAD	Tissues Cell	U	Clinical staging, N classification, M classification, differentiated degree	cell proliferation	Poor prognosis	29367106	10.1016/j.biopha.2018.01.037
LncRNA MACC1-AS1	LUAD	Tissues Cell	U		cell proliferation	Poor prognosis	32109147	10.1089/cbr.2019.3020
LncRNA MUC5B-AS1	LUAD	Tissues Cell Animals	U		Cell migration , invasion,	Poor prognosis	**29670111**	10.1038/s41419-018-0472-6
LncRNA MAFG-AS1	LUAD	Cell	U		cell proliferation and apoptosis	Poor prognosis	**31211984**	10.1016/j.ejphar.2019.172465
LncRNA NEAT1	LUAD	Cell	U		cell proliferation, migration , invasion, apoptosis	Poor prognosis	**30036873**	10.1159/000491958
LncRNA OIP5-AS1	LUAD	Tissues Cell	U	clinical grade	cell proliferation, invasion, migration,	Poor prognosis	29247949	10.1016/j.biopha.2017.12.031
LncRNA PCAT6	LUAD	Tissues Cell	U		cell proliferation, migration、invasion, epithelial-mesenchymal transition(EMT)	Poor prognosis	36787056	10.1007/s11033-023-08259-x
LncRNA RP11-805J14.5	LUAD	Tissues Cell Animals	U		cell growth, migration、invasion	Poor prognosis	35866595	10.3892/or.2022.8376
LncRNA SNHG7	LUAD	Cell Tissues Animals	U	drug resistance	cell proliferation, migration	Poor prognosis	**34715254**	10.1016/j.canlet.2021.10.029
LncRNA SNHG11	LUAD	Tissues Cell Animals	U		cell proliferation、 Cell migration	Poor prognosis	35487027	10.1016/j.prp.2022.153849
LncRNA TTN-AS1	LUAD	Tissues Cell	U		cells proliferation, migration	Poor prognosis	**31173403**	10.1002/jcb.28973
LncRNA TTN - ASI	LUAD	Tissues Cell	U	TNM stage and lymph node metastasis	Cell migration, invasion, and epithelial mesenchymal transition	Poor prognosis	**31363080**	10.1038/s41419-019-1811-y
LncRNA UPLA1	LUAD	Tissues Cell Animals	U	the prognosis and tumor, node, metastasis (TNM) stage	cell proliferation, invasion, migration,	Poor prognosis	33221813	10.1038/s41419-020-03198-y
LncRNA ZXF1	LUAD	Tissues	U	lymph node metastasis, tumor pathological stage	cell migration invasion	Poor prognosis	**24721325**	10.1016/j.biopha.2014.03.001
LncRNA ZXF1	LUAD	Tissues Cell	U	tumor differentiation and lymph node metastasis		Poor prognosis	**28219204**	10.3760/cma.j.issn.0253-3766.2017.02.006
LncRNA ZFPM2-AS1	LUAD	Cell Animals	U		Cell proliferation, invasion epithelial-mesenchymal transition(EMT)	Poor prognosis	31919993	10.1002/1878-0261.12631
LncRNA ZBED5-AS1	LUAD	Tissues Cell Animals	U	inflammatory marker LDH	cell proliferation, migration、 invasion、growth	Poor prognosis	**37762228**	10.3390/ijms241813925
LINC00152	LUAD	Tissues	U	TNM stage, larger tumor size, and lymph node metastasis	cell proliferation	Poor prognosis	28109288	10.1186/s12943-017-0581-3
LINC00426	LUAD	Tissues Cell Animals	U	tumor size, lymphatic metastasis, and tumor differentiation of patients	cell proliferation, migration , invasion, apoptosis	Poor prognosis	**33311443**	10.1038/s41419-020-03259-2
LINC00460	LUAD	Tissues Cell	U		cell proliferation, migration, invasion	Poor prognosis	31789388	10.3892/ijo.2019.4919
LINC00466	LUAD	Cell Animals	U		cell proliferation, migration , invasion, apoptosis	Poor prognosis	**31381886**	10.1016/j.ajpath.2019.06.014
LINC00467	LUAD	Tissues Cell	U		cell proliferation, migration, invasion	Poor prognosis	31180543	10.3892/mmr.2019.10292
LINC00467	LUAD	Cell	U	Tumor sizes and later TNM stages.	cell proliferation, migration	Poor prognosis	**31027730**	10.1016/j.bbrc.2019.04.107
LINC00491	LUAD	Tissues	U	Tumor metastasis and poor survival.	cell proliferation, migration 、invasion	Poor prognosis	36496997	10.3390/cells11233737
LINC00511	LUAD	Tissues Cell	U		cell proliferation, migration, invasion	Poor prognosis	32716147	10.1111/1759-7714.13576
LINC00665	LUAD	Tissues Cell Animals	U	shorter overall survivals	cell growth, invasion	Poor prognosis	34232917	10.18632/aging.203240
LINC00669	LUAD	Cell Animals	U		cell proliferation, apoptosis	Poor prognosis	**36621836**	10.1002/cam4.5604
LINC00707	LUAD	Tissues Cell Animals	U	TNM stage, larger tumor size, lymphatic metastasis, and poor prognosis.	Cell proliferation、migration	Poor prognosis	29482190	10.1159/000487693
LINC00857	LUAD	Tissues Cell Animals	U	Tumor size, TNM stage	cell proliferation, migration , invasion, colony formation	Poor prognosis	**26862852**	10.18632/oncotarget.7203
LINC01123	LUAD	Cell	U		cell proliferation, migration and epithelial-mesenchymal transition (EMT)	Poor prognosis	**33191397**	10.1038/s41419-020-03166-6
LINC01287	LUAD	Cell	U		cell proliferation, apoptosis	Poor prognosis	**34254728**	10.1002/tox.23325
LINC01419	LUAD	Cell	U		cell proliferation, migration、invasion	Poor prognosis	**31582214**	10.1016/j.bbrc.2019.09.090
LINC01426	LUAD	Tissues Cell	U	Tumor diameter; tumor, node, and metastases (TNM) staging; lymph node metastasis (LNM)	cell proliferation, migration、invasion,	Poor prognosis	35266446	10.1080/21655979.2022.2044251
LINC01512	LUAD	Tissues Cell Animals	U	lymph node metastasis and tumor node metastasis (TNM) stage.	Cell proliferation 、adhesion	Poor prognosis	28569418	10.1002/jcb.26178
LINC01977	LUAD	Tissues Cell Animals	U		cell proliferation, invasion	Poor prognosis	35982471	10.1186/s13045-022-01331-2
LINC02418	LUAD	Cell Tissues	U		cell proliferation, migration	Poor prognosis	32795273	10.1186/s12890-020-01229-0

### 4.2 Low-level expressed lncRNA in LUAD and clinical significance

We also analyzed the down-regulation of LncRNAs. In the retrieved data, we found that most of the down-regulation was related to the poor prognosis of patients with lung adenocarcinoma, but the down-regulation of LncRNAs suggested a better prognosis. For example, Liu et al. (21) found that LncRNA SGMS1-AS1 was down-regulated in LUAD tissue as well as cells, which was related to good prognosis of patients with lung adenocarcinoma. The same down-regulation has an effect on cell function. The data are shown in **Table 2**.

**Table 2:** Biological function and clinical significance of down-regulated lncRNA in lung adenocarcinoma.

Order (Low expression)	Pathology type	Sample	Expression	Clinicopathological parameters associated	cell biological function	Prognosis	PMID	DOI
LncRNA ACTA2-AS1	LUAD	Cell	D		cell proliferation, migration, apoptosis ,invasion and epithelial-mesenchymal transition(EMT)	Poor prognosis	**32808728**	10.1002/cbin.11451
LncRNA CARD8-AS1	LUAD	Tissues Cell	D	Tumor size, TNM stage, Lymph node metastasis	cell proliferation, migration and invasion	Poor prognosis	**35996500**	10.3389/bjbs.2022.10498
LncRNA CASC2	LUAD	Tissues Cell	D	TNM stage and large tumor volume	cell proliferation, apoptosis	Poor prognosis	**34337857**	10.1002/kjm2.12386
LncRNA GMDS-AS1	LUAD	Tissues Cell Animals	D	poor survival of LUAD patients.	cell proliferation, migration, invasion, and vivo growth	Poor prognosis	**37309595**	10.2217/epi-2022-0432
LncRNA LMO7DN	LUAD	Tissues Cell	D		cell proliferation	Poor prognosis	**33991849**	10.1016/j.prp.2021.153475
LncRNA LARRPM	LUAD	Tissues Cell Animals	D	shorter survival	cell proliferation, migration, invasion, and apoptosis	Poor prognosis	**36221069**	10.1186/s11658-022-00376-y
LncRNA MIR99AHG	LUAD	Tissues Cell Animals	D		cell proliferation, migration, invasion, glycolysis. vivo growth	Poor prognosis	**36138468**	10.1186/s12967-022-03633-y
LncRNA RPLP0P2	LUAD	Tissues Cell	D		cell migration and adhesion	Poor survival	**27460542**	10.3892/or.2016.4965
LncRNA SGMS1-AS1	LUAD	Tissues Cell	D		cell proliferation, migration , invasion, epithelial-mesenchymal transition(EMT)	Good survival	**34061466**	10.1111/1759-7714.14043
LncRNA SFTA1R	LUAD	Tissues Cell Animals	D		cell migration,	Poor prognosis	**36283586**	10.1016/j.biocel.2022.106317
LncRNA SNHG7	LUAD	Tissues Cell Animals	D		cell proliferation, migration,	Poor prognosis	**32201260**	10.1016/j.ajpath.2020.02.011
LINC00222	LUAD	Tissues Cell	D	larger tumor size, more advanced tumor stage and more frequent lymphatic metastasis	cell proliferation and migration, apoptosis, invasion	Poor prognosis	**29990868**	10.1016/j.biopha.2018.06.165
LINC00365	LUAD	Tissues Cell Animals	D	Tumor size, Histological differentiation, TNM stage, Lymph node metastasis	cell proliferation, migration, invasion, glycolysis and *in vivo* growth	Poor prognosis	35426242	10.1002/tox.23532
LINC00968	LUAD	Tissues Cell Animals	D		cell migration and colony formation	Poor prognosis	**33159015**	10.18632/aging.103833
LINC01089	LUAD	Cell	D		cell proliferation, migration,	Poor prognosis	**34281560**	10.1186/s12890-021-01568-6

### 4.3 High-level expression and low-level expressed LncRNA in LUAD and clinical significance of LncRNA

In the summary, we found that a LncRNA is expressed differently through different pathways in lung adenocarcinoma. It can be either high expression or low expression. However, both high and low expression lead to poor prognosis in patients with lung adenocarcinoma. This lncRNA is SNHG7. Pei et al. (22) found that LncRNA SNHG7 was found to be down-regulated in LUAD tissues compared with normal tissues. And it is related to the poor prognosis of patients. Zhang et al. (23) found that LncRNA SNHG7 was found to be the high-level expression in LUAD. It is related to the poor prognosis of patients and the enhancement of paclitaxel resistance.

## 5 The regulation of signaling pathway that lncRNA participated in LUAD

A large number of studies have proved that LncRNAs are an important regulator of gene regulation, and they can play a key role in a variety of biological functions and disease processes. The LncRNA-mediated gene expression involves various mechanisms, such as regulation of transcription, translation, protein modification, and the formation of RNA-protein or protein-protein complexes (24). Cellular signal transduction plays a key role in various cellular processes that respond to intracellular or extracellular stimuli. Many signaling pathways have been found in cells, which are usually essential for cascading biological responses and gene expression (24). Most studies have shown that LncRNAs are involved in various pathways, and the common signal pathways are P53, Notch, JATT, STAT, PI3K, Akt, Wnt/β-catenin (beta-catenin), and so on.

### 5.1 JAK/STAT signaling pathway

**Figure 2:**
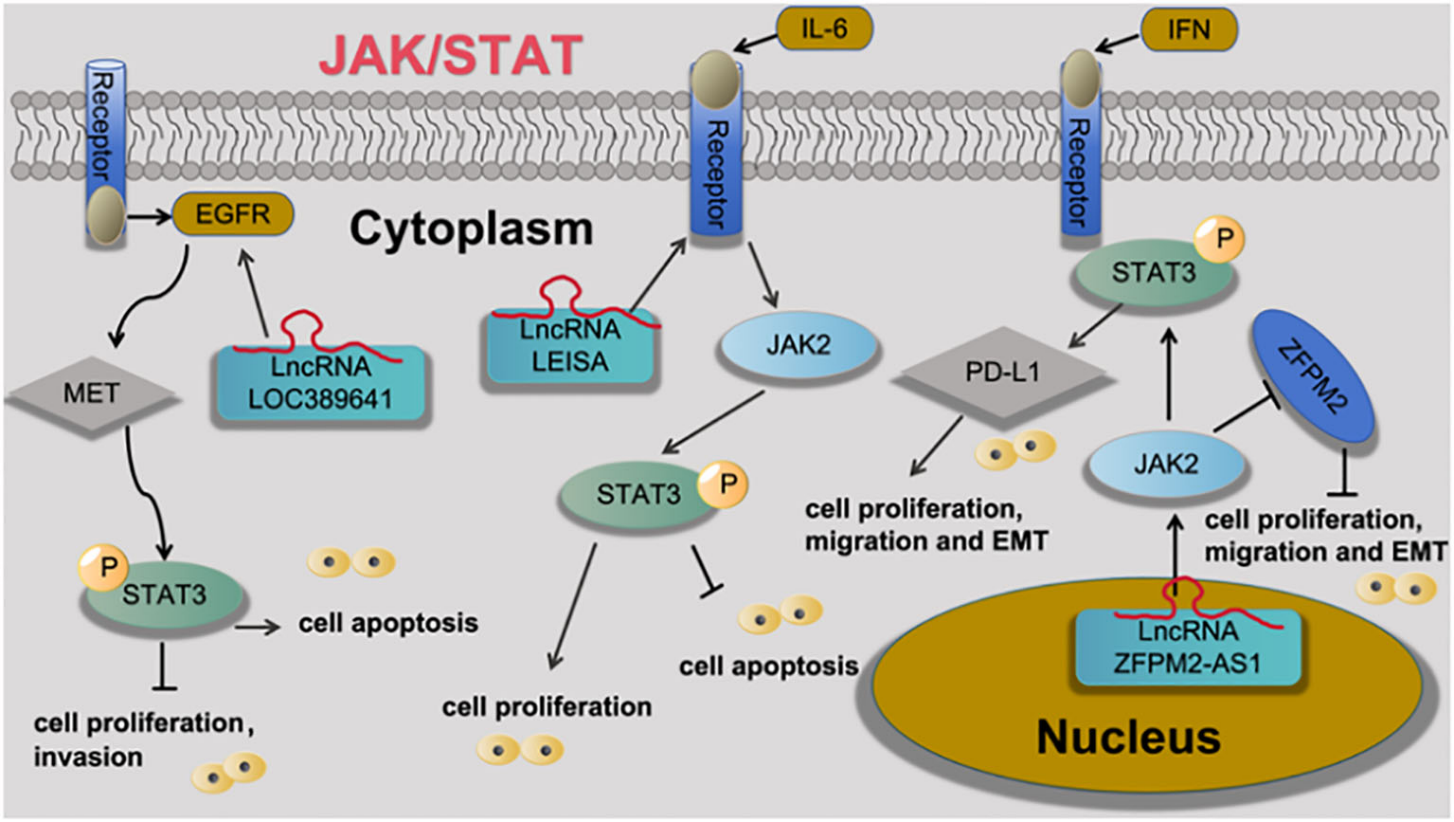
LncRNA is involved in the processes of lung adenocarcinoma cell proliferation, apoptosis, metastasis, invasion and EMT through the regulation of JAK/STAT signaling pathway.

Han et al (25) found that LncRNA ZFPM2-AS1 negative regulation of ZFPM2 expression and positive regulation of PD-L1 expression via the JAK-STAT and AKT pathways. Xiao et al. (26)found that LncRNA LOC389641 is a potential oncogene that acts by influencing MET, EGFR, and STAT3, thereby regulating multiple cell survival/death signals including cell proliferation, colony formation, cell invasion, apoptosis, and autophagy. Wu et al. (27)found that LncRNA LEISA promotes the progression of lung adenocarcinoma via enhancing interaction between STAT3 and IL-6 promoters, as shown in **Figure 2**.

### 5.2 PI3K/Akt/mTOR signaling pathway

**Figure 3:**
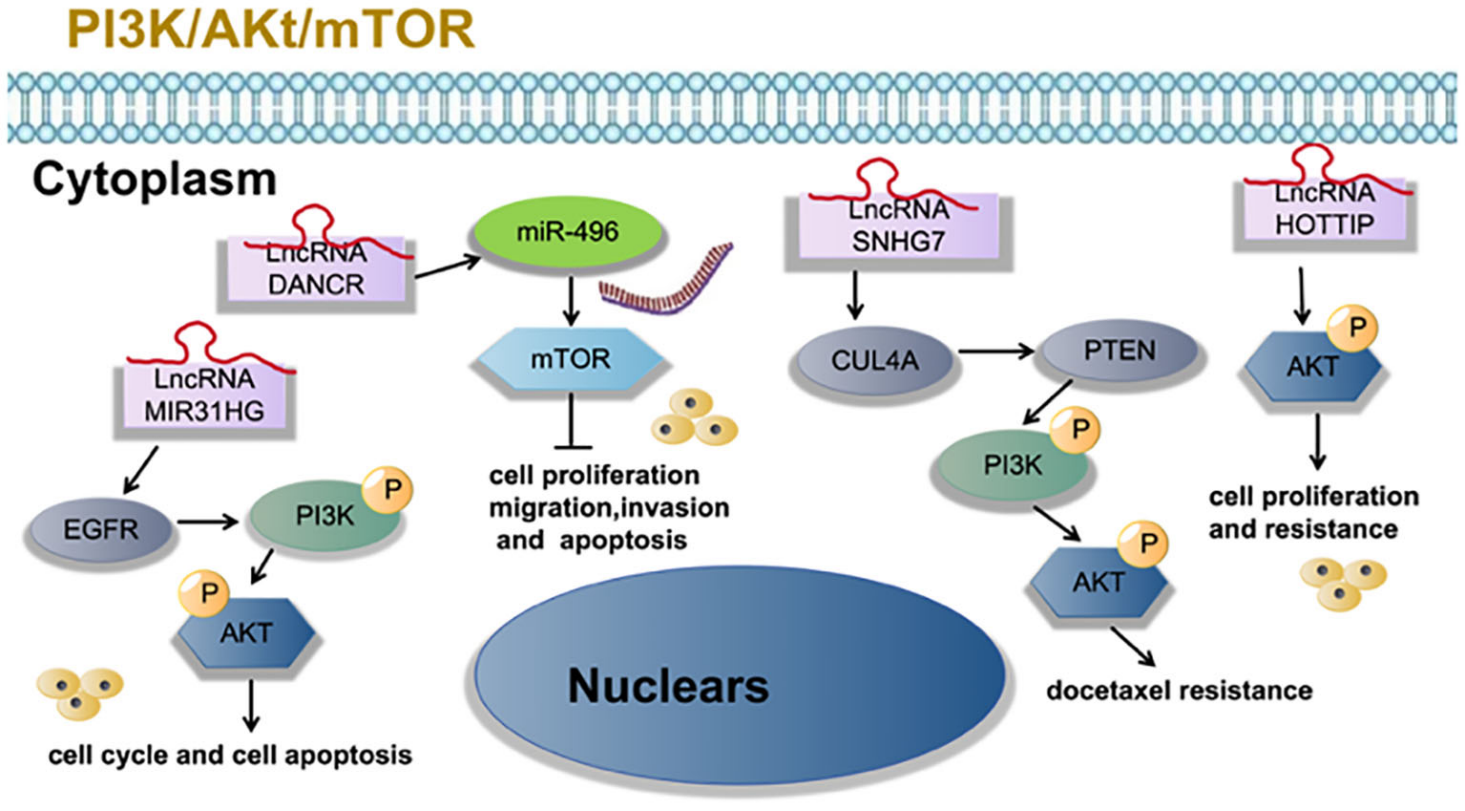
LncRNA is involved in tumor cell proliferation, migration, invasion, apoptosis and other processes of lung adenocarcinoma through PI3K/Akt/mTOR signaling pathway.

Zhang et al. (23) found that LncRNA SNHG7 activates the PI3K/AKT pathway through CUL4A and PTEN, thus enhancing the resistance of LUAD cells to docetaxel resistance. Qin et al. (28) found that LncRNA MIR31HG expression increases gefitinib resistance in non-small cell lung cancer cell lines through the EGFR/PI3K/AKT signaling pathway. Zhang et al. (29) found that overexpression of LncRNA HOTTIP promotes proliferation and drug resistance of lung adenocarcinoma by regulating the AKT signaling pathway. Lu et al. (30) found that LncRNA DANCR contributes to lung adenocarcinoma progression by sponging miR-496 to modulate mTOR expression. as shown in **Figure 3**.

### 5.3 Wnt/β-catenin (beta-catenin) signaling pathway

**Figure 4:**
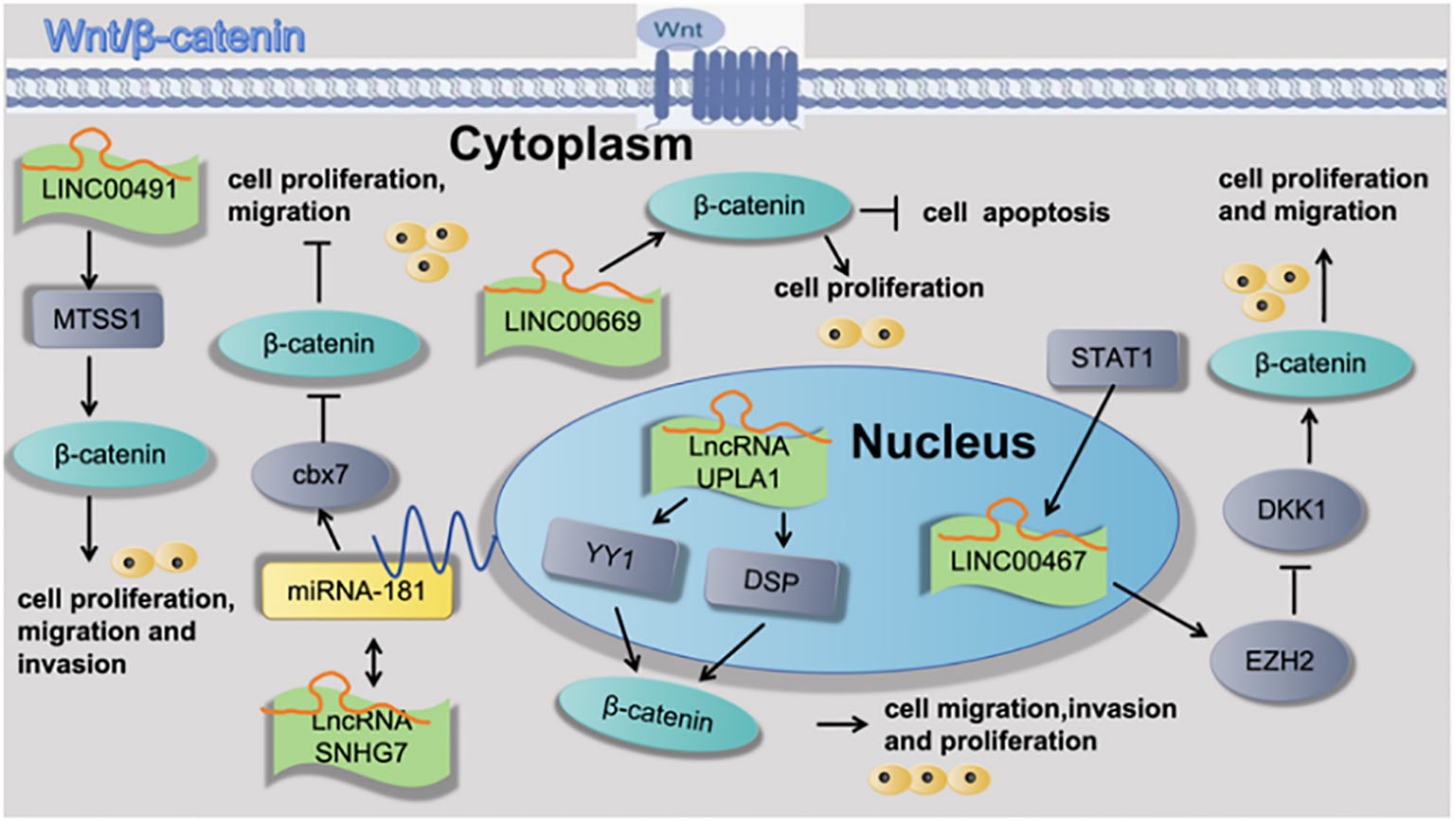
Cell functions of lncRNAs is regulated through the Wnt/β-catenin signaling pathway attributed to the pathogenesis of lung adenocarcinoma.

Han et al. (31) found that LncRNA UPLA1 was mainly located in the nucleus using FISH assay and that it promoted Wnt/β-catenin signaling by binding to desmoplakin using RNA pulldown assay and mass spectrometry. Pei et al. (22) found that LncRNA SNHG7 interacted with microRNA mir-181 and sequentially up-regulated cbx7. cbx7, which suppresses the Wnt/β-catenin pathway in LUAD, was found to be a direct target of mir-181. Yang et al. (32) found that STAT1-induced upregulation of LINC00467 promoted LUAD progression by epigenetically silencing DKK1 to activate the Wnt/β-catenin signaling pathway. Wan et al. (33) found that LINC00491 promotes MTSS1 degradation via the ubiquitin-proteasome degradation through the Wnt/β-Catenin-signaling pathway, and promotes LUAD proliferation and metastasis. Zhu et al. (34) found that LINC00669 promotes lung adenocarcinoma growth by stimulating the Wnt/β-catenin signaling pathway, as shown in **Figure 4**.

### 5.4 P53 signaling pathway

**Figure 5:**
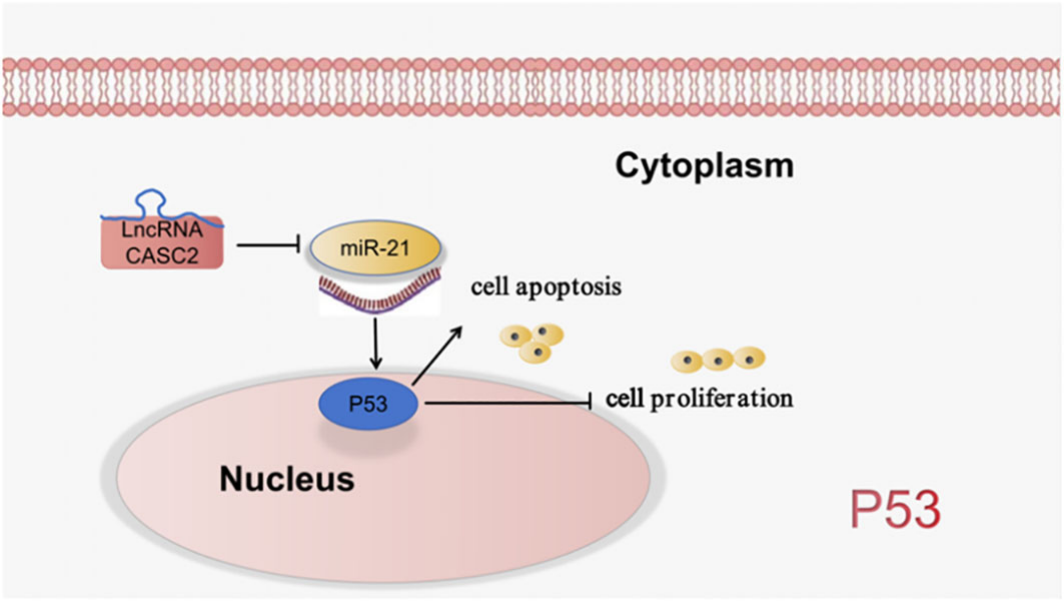
LncRNA is involved in the processes of lung cancer cell proliferation, apoptosis through the regulation of P53 signaling pathway.

Wu et al. (35) found that LncRNA CASC2/miR-21/p53 forms a positive feedback loop to mediate cell proliferation and apoptosis in LUAD, which may provide new insight into the pathological mechanisms of LUAD. As shown in **Figure 5**.

### 5.5 TGF-3/SMAD signaling pathway

**Figure 6:**
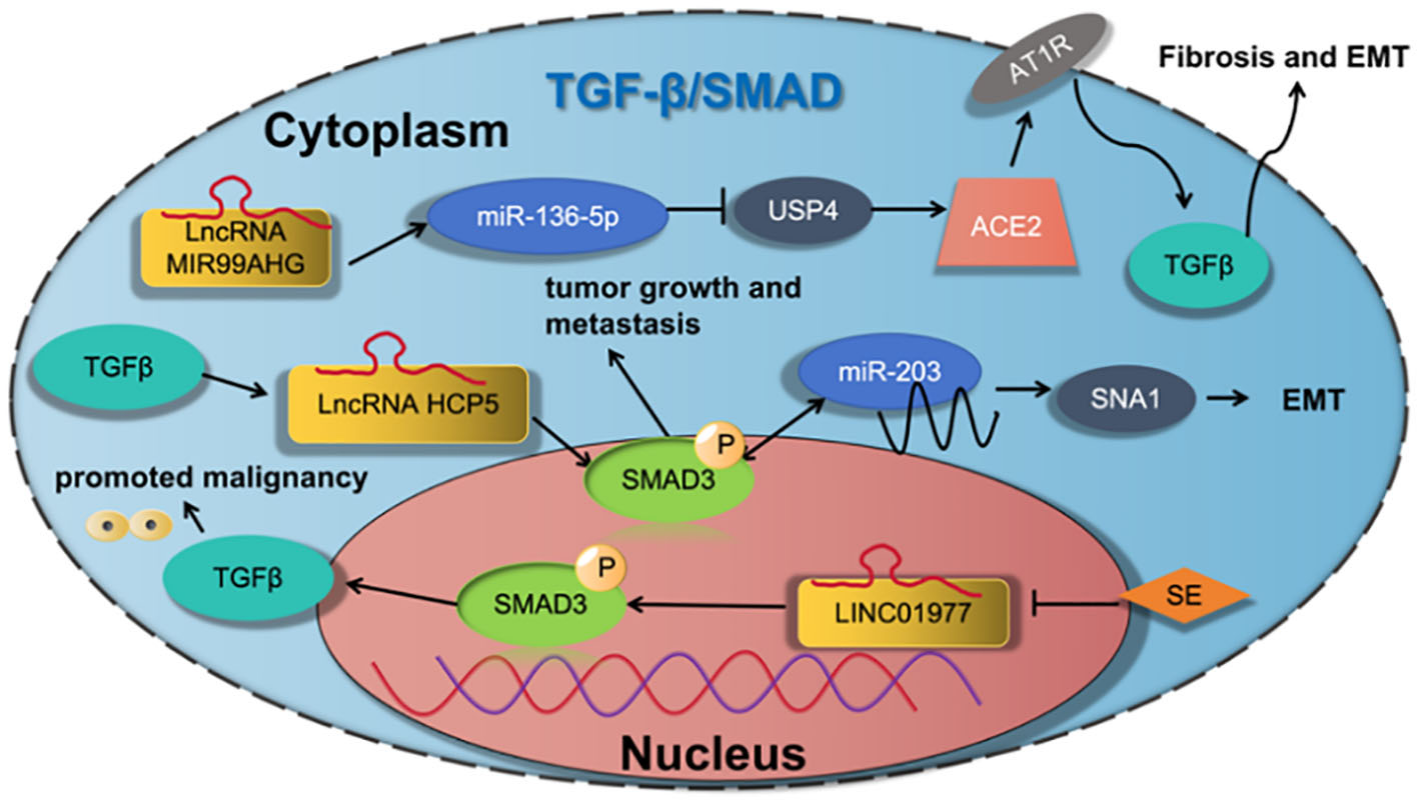
LncRNA regulates tumor motility, invasiveness and other functions of LUAD cells via regulation the TGF-3/SMAD signaling pathway.

Wang et al. (36) found that LncRNA MIR99AHG acted as a sponge of miR-136-5p to inhibit miR-136-5p expression, upregulated the expression of USP4, and enhanced the stability of ACE2 protein, thereby inhibiting EMT in pulmonary fibrosis. Jiang et al. (37) found that LncRNA HCP5 is induced by TGF-β and is directly regulated by SMAD3, which, in turn, is a positive regulator of the TGF-β/SMAD signaling via the *miR-203*/*SNAI* axis. This study demonstrates that overexpression of HCP5 in LUAD is important for promoting EMT and metastasis and may be a potential therapeutic target for LUAD treatment (37). Zhang et al. (38) found that LINC01977, a cancer-testis LncRNA, was hijacked by SE, which promoted proliferation and invasion both *in vitro* and *in vivo*. Activating SMAD3 to bind the promoter and the SE of LINC01977, which up-regulated LINC01977 expression. LINC01977 also promoted malignancy via the canonical TGF-β/SMAD3 pathway. as shown in **Figure 6**.

### 5.6 Notch signaling pathway

**Figure 7:**
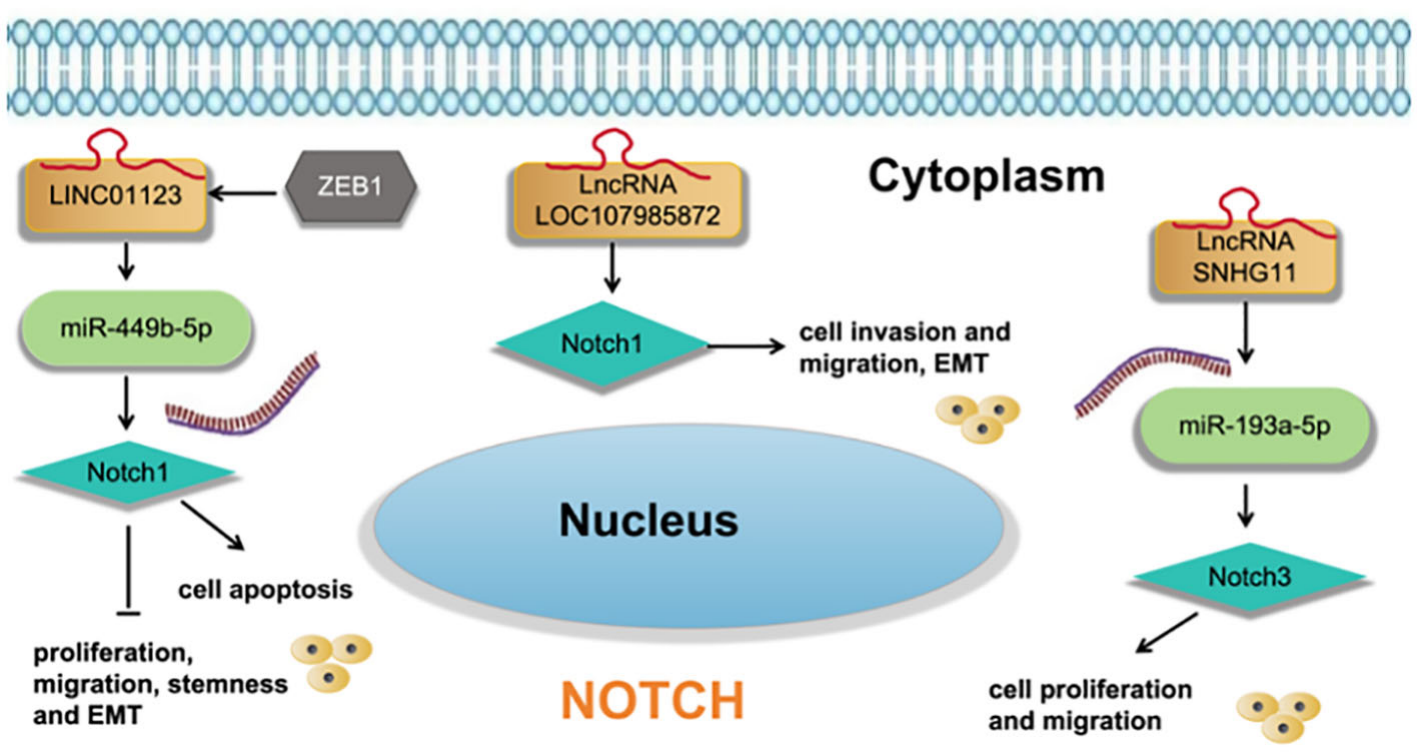
LncRNA regulates cell functions and EMT of LUAD cells via regulation the Notch signaling pathway.

Guo et al. (39) found that LncRNA LOC107985872 promotes lung adenocarcinoma progression via the notch1 signaling pathway with exposure to traffic- originated PM2.5 organic extract. Zhang et al. (40) found that ZEB1-induced LINC01123 mediates LUAD development via miR-449b- 5p/NOTCH1 axis-activated NOTCH1 signaling. Deng et al. (41) found that LncRNA SNHG11 promotes malignant behaviors of LUAD cells by sponging miR-193a-5p and elevating Notch3 expression. as shown in **Figure 7**.

### 5.7 ATR/CHK1 signaling pathway

**Figure 8:**
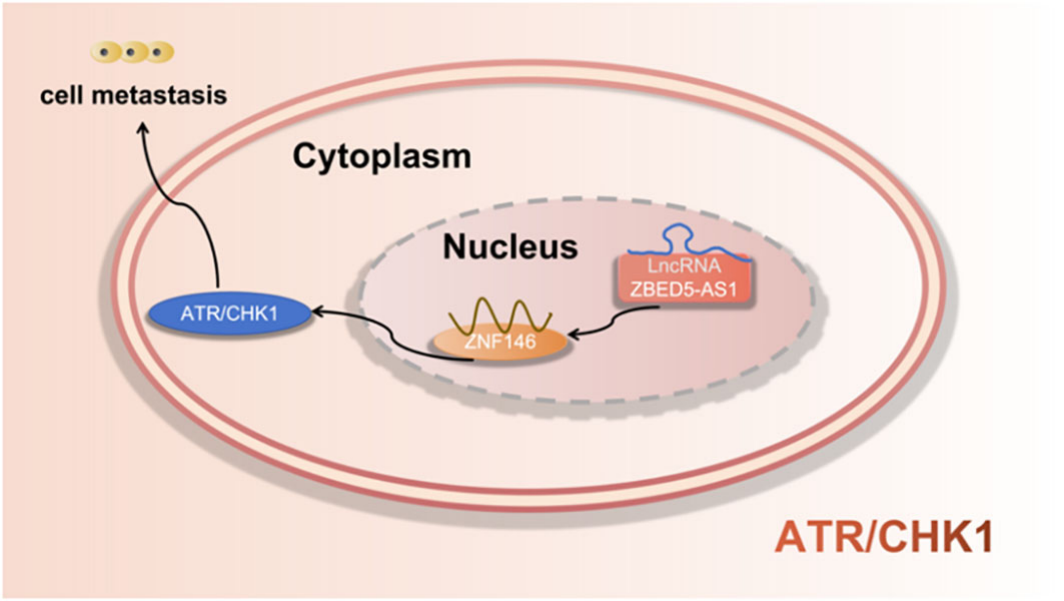
LncRNA regulates metastasis of LUAD cells via regulation the ATRR/CHK1 signaling pathway.

Jiang et al. (42) found that LncRNA ZBED5-AS1 enhanced the metastatic potential of LUAD cells through the EMT pathway by activating ZNF146/ATR/CHK1 axis. as shown in **Figure 8**.

### 5.8 miRNA signaling axis

#### 5.8.1 miRNA-3p

**Figure 9:**
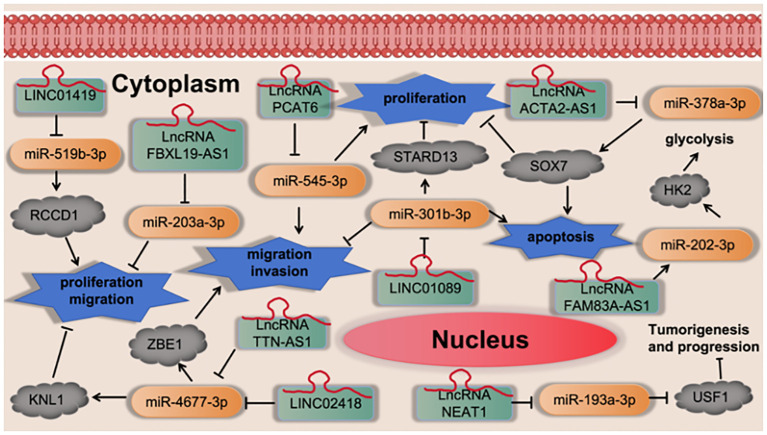
LncRNA regulates cell proliferation, migration, invasion and apoptosis via regulation the miRNA-3p signaling axis.

Cheng et al. (43) found that LINC01419 promotes cell proliferation and metastasis in lung adenocarcinoma via sponging miR-519b-3p to up-regulate RCCD1. Wang et al. (44) found that LncRNA FBXL19-AS1 induces tumor growth and metastasis by sponging miR-203a-3p in lung adenocarcinoma. Yang et al. (45) found that LncRNA PCAT6 promotes proliferation, migration, invasion, and epithelial-mesenchymal transition of lung adenocarcinoma cells by targeting miR-545-3p. Zhong et al. (46) found that LncRNA TTN-AS1 drives the invasion and migration of lung adenocarcinoma cells via modulation of miR-4677-3p/ZEB1 axis. Wang et al. (47) found that LINC02418 promotes malignant behaviors in lung adenocarcinoma cells by sponging miR-4677-3p to upregulate KNL1 expression. Qian et al. (48) found that LINC01089 suppresses lung adenocarcinoma cell proliferation and migration via miR-301b-3p/STARD13 axis. Xiong et al. (49) found that LncRNA NEAT1 may compete with USF1 for binding to miR-193a-3p as a ceRNA and accelerates lung adenocarcinoma deterioration. Ying et al. (50) found that LncRNA ACTA2-AS1 suppresses the malignant processes of LUAD cells by sequestering miR-378a-3p and miR-4428 to augment SOX7 expression. Xiong et al. (51) found that LncRNA FAM83A-AS1 promoted cell migration, invasion, and growth of LUAD cancer cells. Mechanistically, FAM83A-AS1 sponged miR-202-3p to regulate the expression of hexokinase II (HK2) in post-transcription, which facilitated the malignancy and glycolysis. as shown in **Figure 9**.

#### 5.8.2 miRNA-5p of LINC

**Figure 10:**
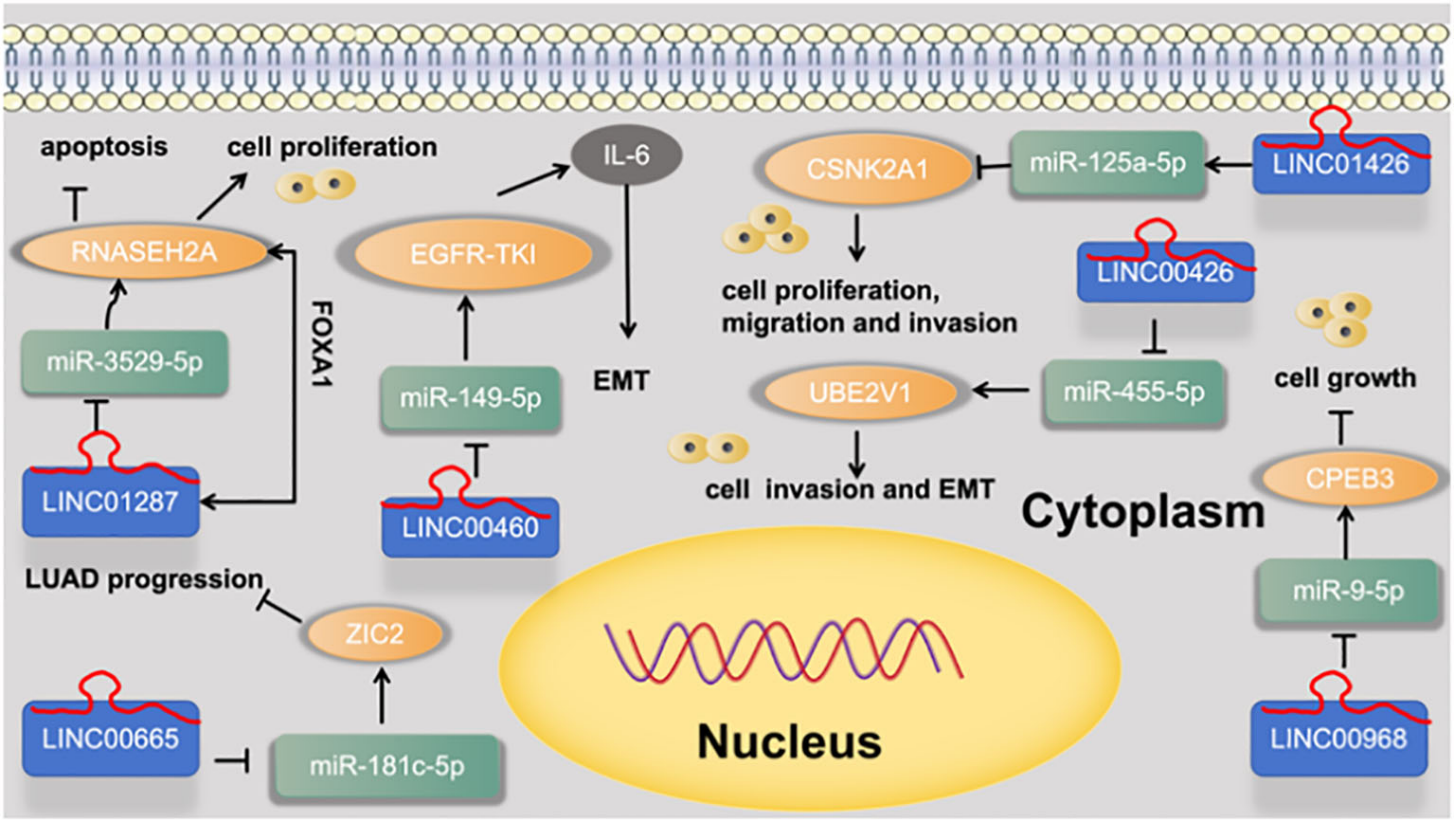
LINC regulates cell functions and EMT via regulation the miRNA-5p signaling axis.

Zhang et al. (52) found that FOXA1-induced LINC01287 serves as a competing endogenous RNA to promote proliferation and inhibit apoptosis of LUAD cells via upregulation of RNASEH2A expression at the posttranscriptional level by competitively combining with miR-3529-5p. Wei et al. (53) found that silencing of LINC00665 suppresses LUAD progression by targeting miR-181c-5p/ZIC2 axis. Yuta Nakano et al. (54) found that LINC00460 was identified as a novel marker of a poor response to EGFR-TKI and prognosis. In lung cancer cells, LINC00460 promoted EGFR-TKI resistance as a competitive decoy for miR-149-5p, thereby facilitating interleukin (IL)-6 expression and inducing EMT-like phenotypes. Li et al. (55) found that LINC00426 accelerates LUAD progression by acting as a molecular sponge to regulate miR-455-5p. Zhu et al. (56) found that LINC01426 promotes the progression of lung adenocarcinoma via regulating miRNA-125a-5p/casein kinase 2 alpha 1 axis. Tang et al. (57) found that LINC00968 inhibits the tumorigenesis and metastasis of lung adenocarcinoma via serving as a ceRNA against miR-9-5p and increasing CPEB3. as shown in **Figure 10**.

#### 5.8.3 miRNA-5p of LncRNA

**Figure 11:**
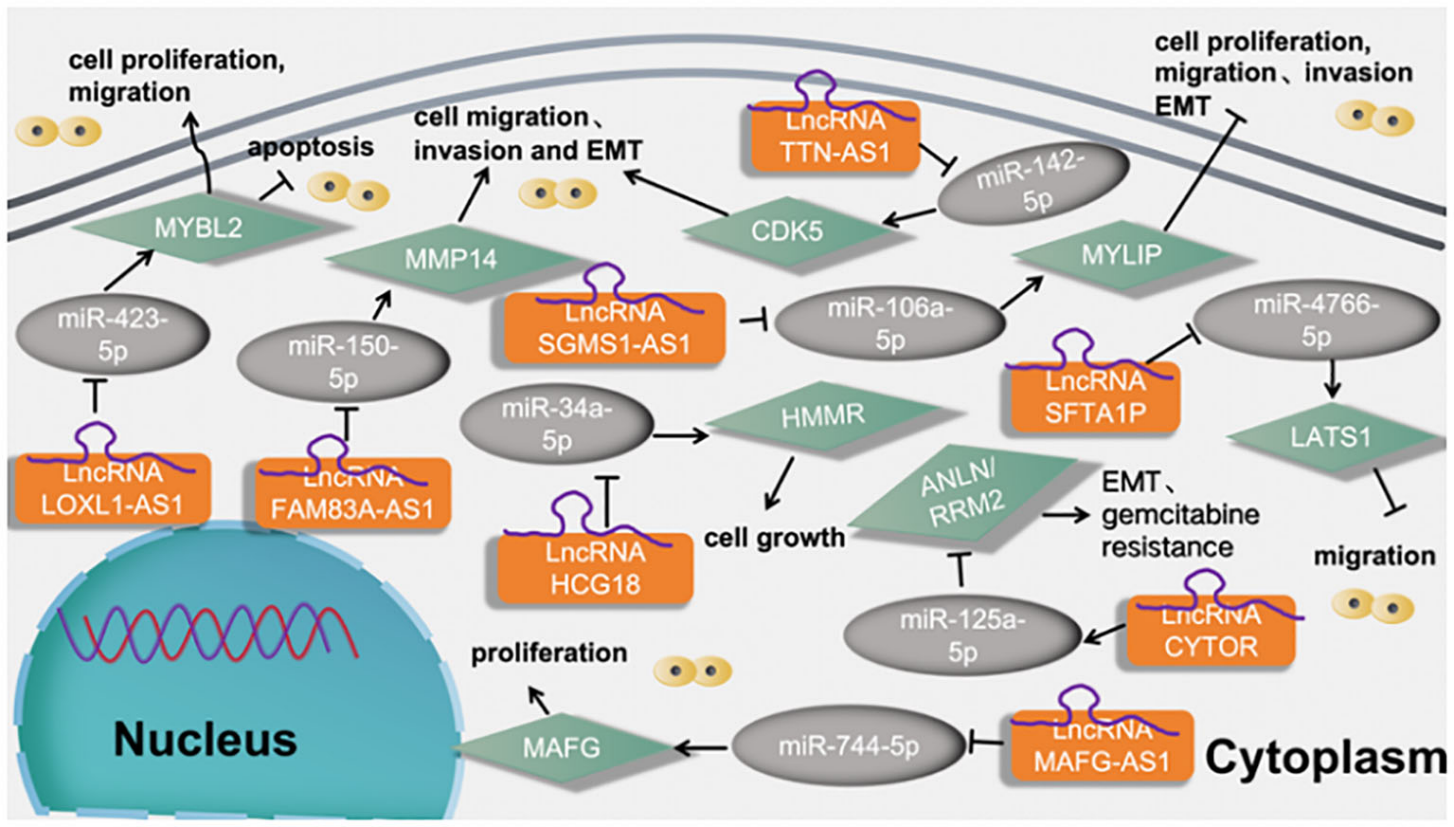
LncRNAs regulates tumor motility and functions of LUAD cells via regulation the miRNA-5p signaling axis.

Xiao et al. (58) found that LncRNA FAM83A-AS1 promotes lung adenocarcinoma cell migration and invasion by targeting miR-150-5p and modifying MMP14. Li et al. (59) found that miR-34a-5p could directly bind to LncRNA HCG18 and miR-34a-5p could directly bind to HCG18. At last, rescue assays proved the carcinogenic role of HCG18/miR-34a-5p/HMMR axis in LUAD cell growth. Liu et al. (21) found that LncRNA SGMS1-AS1 regulates lung adenocarcinoma cell proliferation, migration, invasion, and EMT progression via miR-106a-5p/MYLI9 axis. Jia et al. (60) found that LncRNA TTN-AS1 promotes migration, invasion, and epithelial- mesenchymal transition of lung adenocarcinoma via sponging miR-142-5p to regulate CDK5. Zhang et al. (61) found that LncRNA SFTA1P activates hypoxic exosome-delivered miR-4766-5p through modulating LATS1/YAP pathway, thereby suppressing LUAD cell metastasis, which may serve as a suitable target for the LUAD therapy. Sui et al. (62) found that LncRNA MAFG-AS1 served as a molecular sponge of miR-744-5p to upregulate its nearby gene MAFbZIP transcription factor G (MAFG) in LUAD cells. Finally, LncRNA MAFG-AS1 boosts the proliferation of lung adenocarcinoma cells via regulating miR-744-5p/MAFG axis. Cao et al. (63) found that LncRNA CYTOR upregulates ANLN and RRM2 proteins by inhibiting miR-125a-5p, thus promoting gemcitabine resistance and EMT in lung adenocarcinoma. Li et al. (64) found that LncRNA LOXL1‐AS1 functioned as an oncogene in LUAD, and regulated LUAD development through sponging miR‐423‐5p and targeting MYBL2. as shown in **Figure 11**.

## 6 Conclusions

Lung cancer ranks second among health problems and is the major cause of cancer-associated mortality worldwide. LUAD is considered to be the most common subtype in nonsmokers. The prevalence of LUAD is rapidly increasing with the development of anti-smoking movements. Although headway has been made in cancer treatment, the overall survival of LUAD remains disappointing due to a lack of reliable early prognostic indicators (65). LncRNAs, over the last decade, have been recognized as a diverse class of macromolecules in terms of function and mechanism. Many LncRNAs have demonstrated functional activity in a wide range of cancers. While the LncRNAs transcriptome is far from complete, we now have an appreciation of their diversity due to cell-type specificity. This characteristic can aid in the development of early detection methods and targeted therapies for multiple cancer types. Alongside these immediate applications, understanding the mechanism(s) by which these transcripts are regulated will shed light on the etiology of cancer development, allowing clinicians to implement better treatment strategies and improve overall survival rates. Given the effect of differential expression of LncRNAs on the progression of lung adenocarcinoma, we looked up a large number of literature and analyzed it. We found that the high and low expression of LncRNAs can promote or inhibit lung adenocarcinoma cells, mainly affecting tumor proliferation, migration, apoptosis, and invasion. At the same time, for the treatment of anticancer drugs, LncRNAs will affect the drug resistance of patients with lung adenocarcinoma to anticancer drugs. At present, lung adenocarcinoma is one of the major cancers threatening the lives of Chinese people. We need to pay a lot of attention to the study of lung adenocarcinoma to improve the prognosis and survival rate of patients. It has important clinical significance for the detection of tumor markers of lung adenocarcinoma and the improvement of early detection, early diagnosis, and early treatment of patients with lung adenocarcinoma. The main purpose of this paper is to summarize the effects of LncRNAs on the occurrence and development of lung adenocarcinoma and to provide a new idea for future research on lung adenocarcinoma-related LncRNAs. About the study of LncRNAs in lung adenocarcinoma, there are still many deficiencies in our research, which will be further studied in the future.

## Author contributions

HW: Conceptualization, Writing – review & editing, Funding acquisition. SZ: Writing – original draft. XL: Data curation, Formal analysis, Writing – review & editing. RF: Data curation, Formal analysis, Writing – review & editing. LL: Conceptualization, Formal analysis, Writing – review & editing, Funding acquisition.
